# Catalog of the adelgids of the world (Hemiptera, Adelgidae)

**DOI:** 10.3897/zookeys.534.6456

**Published:** 2015-11-11

**Authors:** Colin Favret, Nathan P. Havill, Gary L. Miller, Masakazu Sano, Benjamin Victor

**Affiliations:** 1 University of Montreal, Biodiversity Centre, 4101 rue Sherbrooke est, Montreal, Quebec, H1X 2B2 Canada; 2 USDA Forest Service, Northern Research Station, 51 Mill Pond Road, Hamden, CT 06514, USA; 3 USDA-ARS, Systematic Entomology Laboratory, 10300 Baltimore Ave, Bldg. 005, BARC-West, Beltsville, MD 20705, USA; 4 Hokkaido University, Graduate School of Agriculture, Systematic Entomology, Kita 9 Nishi 9, Kita-ku, Sapporo 060-8589, Hokkaido, Japan; 5 University of Montreal, Classical Studies Centre, 3774 rue Jean-Brillant, Montreal, Quebec, H3T 1P1 Canada

**Keywords:** Aphidomorpha, nomenclature, Sternorrhyncha, taxonomy, woolly adelgid

## Abstract

A taxonomic and nomenclatural Catalogue of the adelgids (Hemiptera: Adelgidae) is presented. Six family-group names are listed, five being synonyms of Adelgidae. Twenty-two genus-group names, of which nine are subjectively valid and in use, are presented with their type species, etymology, and grammatical gender. One hundred and six species-group names are listed, of which 70 are considered subjectively valid.

## Introduction

Adelgidae is a small family of Hemiptera with 65 species, closely related to Aphididae. They exhibit a two-year life cycle, with some species alternating hosts between spruce (*Picea*) one year and species of another conifer genus (*Abies*, *Larix*, *Pinus*, *Pseudotsuga*, *Tsuga*) the next. Other species or populations do not alternate hosts, feeding only on *Picea* or one of the other conifer genera. Like other Aphidomorpha, Adelgidae exhibit cyclical parthenogenesis, although they are oviparous unlike the viviparous Aphididae. Some adelgid species are important forestry pests, most notably the hemlock woolly adelgid, *Adelges tsugae* (Annand) and the balsam woolly adelgid, *Adelges piceae* (Ratzeburg). Havill and Foottit (2007) present a thorough overview of the biology and evolution of the family.

The closest relatives of the Adelgidae are two extinct families, Elektraphididae and Mesozoicaphididae, the three families comprising the superfamily Adelgoidea (Heie and Wegierek 2009). The oldest fossils of the extinct families date to the Cretaceous, whereas the only fossil adelgid, *Adelges balticus* Wegierek, 2003, is Eocene in age. Heie and Wegierek (2011) present a list of the fossil Aphidomorpha, including the one adelgid and the 20 other Adelgoidea species.

Adelgid classification has long been unstable. It was first hampered by the adoption of the genus name *Chermes* Linnaeus, 1758, which had also been used for psyllids and scale insects (Favret et al. 2014). The name was eventually suppressed by the International Commission on Zoological Nomenclature (Evans and China 1965), but not before 137 nominal species had been described in combination with *Chermes*, including 43 Adelgidae (Favret et al. 2014). Towards the end of the 19^th^ Century and in the first half of the 20^th^ Century, several workers described many new species and erected new genera. Most notably, there was little agreement about adelgid taxonomy between the two most prolific adelgid workers, the Russian N.A. Cholodkovsky and the German C. Börner. With the application of molecular data and explicit systematic analyses (Havill et al. 2007, Havill and Foottit 2007), adelgid classification has become more stable. Many researchers have adopted a system with two genera (*Adelges* Annand and *Pineus* Shimer) and several subgenera, although some have preferred to treat the latter as full genera (Binazzi 1984, Zurovcová et al. 2010).

While several world catalogs and species lists of the more diverse Aphididae have been published (Wilson and Vickery 1918, Patch 1938, Hille Ris Lambers and Eastop 1976, Remaudière and Remaudière 1997), a comprehensive catalog of the Adelgidae has never been produced. Perhaps the small size of the family contributed to its neglect, but the relative inaccessibility of the taxonomic literature, written in multiple languages and in often hard-to-find sources, possibly made the task too daunting. We here present a taxonomic and nomenclatural list of the extant adelgid nominal taxa, including six family-group, 22 genus-group, and 106 species-group names. Of these, we list one valid family (Adelgidae), two and seven valid genera and subgenera (not counting nominotypical subgenera), and 65 and five valid species and subspecies (not counting nominotypical subspecies). We include one genus-group nomen dubium, four suppressed genus-group names, three species-group nomina dubia, and two unavailable species-group names; the many such names still in combination with *Chermes* are not listed here (Favret et al. 2014).

Despite having nomenclatural priority, *Coccus laricis* Bouché, 1834 has long been treated as a synonym of *Adelges laricis* Vallot, 1836 (Börner 1952, Steffan 1972, Eastop and Hille Ris Lambers 1976). *Coccus laricis* Bouché has not been used as a valid name after 1899, meeting the requirements of nomen oblitum per ICZN Article 23.9.1.1 (International Commission on Zoological Nomenclature 1999). Meanwhile, *Adelges laricis* Vallot has been used frequently. To address ICZN Article 23.9.1.2 and thus consider *Adelges laricis* Vallot a nomen protectum, we here list 25 works that used the name as valid, published by at least ten authors in the immediately preceding 50 years and encompassing a span of not less than ten years (Carter 1971, Podeur 1971, Steffan 1972, Li and Tsai 1973, Parry 1973, Eichhorn and Carter 1978, Zhang et al. 1980, Fang 1982, Binazzi 1984, Luo 1988, Rohfritsch 1988, Eichhorn 1994, Battisti et al. 1997, Fang and Yan 1997, Szklarzewicz et al. 2000, Wegierek 2002, Rożkowski 2004, Skrzypczyńska 2004, Havill and Foottit 2007, Havill et al. 2007, Zurovcová et al. 2010, Michalik et al. 2012, Sano and Ozaki 2012, Toenshoff et al. 2012, Gavrilov-Zimin et al. 2015).

In order to facilitate future species descriptions, we followed the model of recent aphid genus-group catalogs by including information on etymology and grammatical gender (Favret et al. 2008, 2009, Cortés Gabaudan et al. 2011, Nieto Nafría et al. 2011). In cases where two page numbers are provided for original descriptions, the first number refers to a nomenclaturally valid diagnosis, such as in a dichotomous key, the second references the formal description. Valid names are in bold font, synonyms are preceded by ‘=’. Synonyms of family-group names are presented with their rank-specific endings replaced by ‘__’. Nominal species are listed under their current generic placement with the original genus in parentheses. The taxonomic catalog is followed by an alphabetical index to help find the current placement of each name. It is our hope that this catalog will serve as an initial point of convergence in our understanding of adelgid systematics and a point of departure on which future research will be built. Future updates will be published on Aphid Species File (Aphid.SpeciesFile.org).

## Catalogue

**ADELGIDAE**Annand 1928:31

Original spelling. Adelginae

Type genus. *Adelges*Vallot 1836

=CHERMES__ Herrich-Schaeffer 1854:VIII

Original spelling. Chermiden

Type genus. *Chermes*Linnaeus 1758

Note. Suppressed (Evans and China 1965)

=CHERMAPH__ Hunter 1901:70

Original spelling. Chermaphinae

Type genus. *Chermaphis*Maskell 1884

=DREYFUSI__ Börner 1930:155

Original spelling. Dreyfusiini

Type genus. *Dreyfusia*Börner 1908a

=PINE__ Nüsslin in Börner 1909c:51

Original spelling. Pineini

Type genus. *Pineus*Shimer 1869

=SACCHIPHANT__ Steffan 1968:128

Original spelling. Sacchiphantini

Type genus. *Sacchiphantes*Curtis 1844

***ADELGES***Vallot 1836

Subgenus ***ADELGES***Vallot 1836:72

Type species. *Adelges laricis*Vallot 1836, by original monotypy

Etymology. Greek adelos ‘unclear’, ‘secret’ + Greek ge ‘earth’ + -s [concealed in the earth]

Gender. Masculine

=*ANISOPHLEBA*Koch 1857:320

Type species. *Anisophleba hamadryas*Koch 1857, by subsequent monotypy

Etymology. Greek anisos ‘unequal’ + Greek phleps ‘vein’ + -a

Gender. Feminine

=*LARICETHUS*Amyot 1847:485

Type species. *Adelges laricis*Hartig 1839, by original monotypy

Etymology. Greek larix ‘larch’ + Greek eth- ‘custom’, ‘habit’ + -us

Gender. Masculine

Note. Suppressed (China 1963)

***aenigmaticus***Annand 1928:34,73 (*Adelges*)

***geniculatus*** (Ratzeburg 1844:202) (*Chermes*)

***isedakii*** Eichhorn in Eichhorn and Carter 1978:280 (*Adelges*)

***japonicus*** (Monzen 1929:71) (*Chermes*)

***karamatsu***Inouye 1945:54,86 (variety of *Adelges laricis* Vallot)

***lapponicus*** (Cholodkovsky 1889b:390) (*Chermes*)

=*praecox* (Cholodkovsky 1898:28) (variety of *Chermes lapponicus* Cholodkovsky)

***lariciatus*** (Patch 1909:137) (*Chermes*)

***laricis***Vallot 1836:72 (*Adelges*) (nomen protectum with respect to *Coccus laricis* Bouché)

subspecies ***laricis***Vallot 1836

=*atratus* (Buckton 1883:39) (*Chermes*)

=*coccineus* (Ratzeburg 1843:202) (*Chermes*)

=*consolidatus* (Patch 1909:137) (*Chermes*)

=*hamadryas* (Koch 1857:320) (*Anisophleba*)

=*lariceti* (Altum 1889:279) (*Chermes*)

=*laricis* (Bouché 1834:22) (*Coccus*) (nomen oblitum with respect to *Adelges laricis* Vallot)

=*laricis* (Hartig 1839:644) (*Chermes*)

=*obtectus* (Ratzeburg 1844:200) (*Chermes*)

=*strobilobius* (Kaltenbach 1843:203) (*Chermes*)

subspecies ***potaninilaricis*** Zhang in Zhang et al. 1980:383 (subspecies of *Adelges laricis* Vallot)

***tardoides*** (Cholodkovsky 1911:175) (variety of *Chermes strobilobius* Kaltenbach)

***tardus*** (Dreyfus 1888:3) (*Chermes*)

=*affinis* (Börner 1908b:167) (*Cnaphalodes*) (Börner 1908a:417 nomen nudum)

=*niger* (Solowiow 1924:41) (*Chermes*)

Subgenus ***ANNANDINA***Favret et al. 2015:176

Type species *Adelges tsugae*Annand 1924b, by original designation

Etymology. (Percy Nicol) Annand [American entomologist] + -ina

Gender. Feminine

***tsugae***Annand 1924b:79 (*Adelges*)

Subgenus ***APHRASTASIA***Börner 1909a:1

Type species. *Chermes pectinatae*Cholodkovsky 1888, by original monotypy

Etymology. Greek aphrastos ‘unnoticed’ + -ia

Gender. Feminine

***pectinatae*** (Cholodkovsky 1888:47) (*Chermes*)

subspecies ***ishiharai*** (Inouye 1936:75) (*Chermes*)

subspecies ***pectinatae*** (Cholodkovsky 1888)

Subgenus ***CHOLODKOVSKYA***Börner 1909b:1

Type species. *Chermes viridanus*Cholodkovsky 1896, by original monotypy

Etymology. (Nikolai Alexandrovitsch) Cholodkovsky [Russian entomologist] + -a

Gender. Feminine

***oregonensis***Annand 1928:34,67 (*Adelges*)

***viridanus*** (Cholodkovsky 1896:39) (*Chermes*)

=*laricicola* (Shinji 1930:151) (*Chermes*)

***viridulus*** (Cholodkovsky 1911:175) (*Chermes*)

Subgenus ***DREYFUSIA***Börner 1908a:416

Type species. *Chermes piceae*Ratzeburg 1844, by subsequent designation (Börner 1908b:138)

Etymology. (Ludwig Theodor) Dreyfus [German entomologist] + -ia

Gender. Feminine

*abietispiceae* (Stebbing 1903:57) (*Chermes*) nomen dubium (Choldkovsky (1906:49, 1915:60) indicates that the species is probably real but that the description is insufficient to confirm its identity; Schneider-Orelli and Schneider (1954:414) agree that Stebbing’s description provides insufficient morphological information to compare with their description of *Dreyfusia knucheli* Schneider-Orelli and Schneider. Ghosh (1983:11) considered it a synonym of *Dreyfusia knucheli*, but qualified it with a question mark)

=*himalayensis* (Stebbing 1910:100) (*Chermes*) (unavailable, described in synonymy with *Chermes abietispiceae*Stebbing 1903)

*funitectus* (Dreyfus 1888:6) (*Chermes*) nomen dubium (Favret et al. 2015)

***joshii*** (Schneider-Orelli and Schneider 1959:260) (*Dreyfusia*)

***knucheli*** (Schneider-Orelli and Schneider 1954:416) (*Dreyfusia*)

***merkeri*** (Eichhorn 1957:312) (*Dreyfusia*)

***nebrodensis*** (Binazzi and Covassi 1991:246) (*Dreyfusia*)

***nordmannianae*** (Eckstein 1890:90) (*Chermes*)

=*nuesslini* (Börner 1908c:739) (*Dreyfusia*)

***piceae*** (Ratzeburg 1844:204) (*Chermes*)

subspecies ***canadensis*** (Merker and Eichhorn 1956:454) (form of *Dreyfusia piceae* (Ratzeburg))

subspecies ***occidentalis***Foottit and Mackauer 1983:302 (subspecies of *Adelges piceae* (Ratzeburg))

subspecies ***piceae*** (Ratzeburg 1844)

=*bouvieri* (Cholodkovsky 1902:10) (variety of *Chermes piceae* Ratzeburg)

***pindrowi***Yaseen and Ghani 1971:191,193 (*Adelges*)

***prelli*** (Grosmann 1935:822) (*Dreyfusia*)

***schneideri*** (Börner 1931:684) (*Dreyfusia*)

***todomatsui*** (Inouye 1945:73,92) (*Dreyfusia*)

Subgenus ***GILLETTEELLA***Börner 1930:157

Type species. *Chermes cooleyi*Gillette 1907, inherited from replaced name

Etymology. (Clarence Preston) Gillette [American entomologist] + -ella [diminutive suffix]

Gender. Feminine

Note. Replacement name for *Gillettea*Börner 1909d

=*GILLETTEA*Börner 1909d:504

Type species. *Chermes cooleyi*Gillette 1907, by original monotypy

Etymology. (Clarence Preston) Gillette [American entomologist] + -a

Gender. Feminine

Note. Junior homonym of *Gillettea*Ashmead 1897:69 (Hymenoptera, Cynipidae)

***cooleyi*** (Gillette 1907:3) (*Chermes*)

***coweni*** (Gillette 1907:10) (variety of *Chermes cooleyi* Gillette)

*cummingae* (Steffan 1968:10,22,42) (*Gilletteella*) nomen nudum

***glandulae*** (Zhang in Zhang et al. 1980:382) (*Gilletteella*)

Subgenus ***SACCHIPHANTES***Curtis 1844:831

Type species. *Chermes abietis*Linnaeus 1758, by original monotypy

Etymology. Greek sakkos ‘coarse cloth’, ‘sail’ + Greek hyphantēs ‘weaver’

Gender. Masculine

=*ELATIPTUS*Amyot 1847:484

Type species. *Chermes abietis*Linnaeus 1758, by original monotypy

Etymology. Greek elatē ‘fir’ + Greek ipt- ‘harm’ + -us

Gender. Masculine

Note. Suppressed (China 1963) and junior objective synonym of *Sacchiphantes*Curtis 1844

=*PHLOEOPHTHIRIDIUM*van der Hoeven 1849:509

Type species. *Chermes abietis*Linnaeus 1758, by original monotypy

Etymology. Greek phloios ‘bark’ + Greek phtheir- ‘destroy’ + Greek -idium [diminutive suffix]

Gender. Neuter

Note. Junior objective synonym of *Sacchiphantes*Curtis 1844

***abietis*** (Linnaeus 1758:454) (*Chermes*)

=*abietislaricis* (Eckstein 1890:88) (*Chermes*) based on Dreyfus’s (1889:82) description of a *Larix*-associated form of *Chermes abietis*

=*alaeviridis* (Solowiow 1924:39) (*Chermes*)

=*gallarumabietis* (De Geer 1773:99) (*Aphis*) nomen dubium

=*laricifoliae* (Fitch 1858:752) (*Chermes*)

***diversis***Annand 1928:34,69 (*Adelges*)

***karafutonis***Kono and Inouye 1938:169,170 (*Adelges*)

***kitamiensis*** (Inouye 1963:341) (*Sacchiphantes*)

***roseigallis*** (Li and Tsai 1973:135,141,148) (*Sacchiphantes*)

***segregis*** (Steffan 1961:67) (*Sacchiphantes*)

***torii*** (Eichhorn in Eichhorn and Carter 1978:284) (*Sacchiphantes*)

***viridis*** (Ratzeburg 1843:202) (*Chermes*)

=*occidentalis* (Cholodkovsky 1910:281) (*Chermes*)

*CHERMES*Linnaeus 1758:453

Type species. None (see Favret et al. 2014)

Etymology. Arabic kirmiz ‘crimson’

Gender. Masculine

Note. Suppressed (Evans and China 1965), see Favret et al. (2014) for list of species-group names described under *Chermes*

*GISTELIELLA*Strand 1928:46

Type species. *Chermes lapidarius*Fabricius 1803, inherited from replaced name

Etymology. (Johannes) Gistel [German entomologist] + -i + ella [diminutive suffix]

Gender. Feminine

Note. Replacement name for *Aphanus*Gistel 1837, nomen dubium

=*APHANUS*Gistel 1837:111

Type species. *Chermes lapidarius*Fabricius 1803, by original monotypy

Etymology. Greek aphanēs ‘invisible’

Gender. Masculine

Note. Junior homonym of *Aphanus*Laporte 1833:35 (Hemiptera, Lygaeidae)

*lapidaria* (Fabricius 1803:306) (*Chermes*) nomen dubium (placement unknown)

***PINEUS***Shimer 1869:383

Subgenus ***PINEODES***Börner 1926:240

Type species. *Chermes pinifoliae*Fitch 1858, by original monotypy

Etymology. *Pine(us)* [Hemiptera: Adelgidae]+ Greek -ōdēs ‘resembling’

Gender. Masculine

***pinifoliae*** (Fitch 1858:741) (*Chermes*)

=*abieticolens* (Thomas 1879:156) (*Chermes*)

=*armiger* (Annand 1924a:5) (*Chermes*)

=*montanus* (Gillette 1907:14) (*Chermes*)

Subgenus ***PINEUS***Shimer 1869:383

Type species. *Coccus pinicorticis*Fitch 1855, by original monotypy

Etymology. Latin pineus ‘of or pertaining to pine’

Gender. Masculine

=*CHERMAPHIS*Maskell 1884:292

Type species. Kermaphis pini var. laevisMaskell 1885, by subsequent designation (Eastop and Hille Ris Lambers 1976:144)

Etymology. *Cherm(es)* [Hemiptera] + *Aphis* [Hemiptera: Aphididae]

Gender. Feminine

=*CNAPHALODES*Amyot and Audinet Serville 1843:595

Type species. *Chermes pini*Goeze 1778, by original monotypy

Etymology. Greek knaphallon ‘pillow’ + Greek -ōdēs ‘resembling’

Gender. Masculine

=*EOPINEUS*Steffan 1968:11

Type species. *Chermes strobi*Ratzeburg 1844, by subsequent designation (Eastop and Hille Ris Lambers 1976:188)

Etymology. Greek ēōs ‘dawn’, ‘early’ + *Pineus* [Hemiptera: Adelgidae]

Gender. Masculine

=*KERMAPHIS*Maskell 1885:19

Type species. *Anisophleba pini*Koch 1857, by subsequent designation (Eastop and Hille Ris Lambers 1976:232)

Etymology. *Kerm(es)* [Hemiptera: Coccoidea] + *Aphis* [Hemiptera: Aphididae]

Gender. Feminine

=*PITYOPSYLLA*Amyot 1847:461

Type species. *Chermes pini*Goeze 1778, by original monotypy

Etymology. Greek pitys ‘pine’ + *Psylla* [Hemiptera: Psyllidae]

Gender. Feminine

Note. Suppressed (China 1963) and junior objective synonym of *Cnaphalodes*Amyot and Audinet Serville 1843

***abietinus***Underwood and Balch 1964:523 (*Pineus*)

***armandicola***Zhang et al. 1992:360,394 (*Pineus*)

***boerneri***Annand 1928:34,112 (*Pineus*) (Takahashi (1937:11), Inouye (1945:36), and Ghosh (1983:8) list this species as a synonym of *Pineus laevis* (Maskell), but Maskell’s (1885:16) description is insufficient to validate synonymy)

***boycei***Annand 1928:35,102 (*Pineus*)

***cembrae*** (Cholodkovsky 1888:47) (*Chermes*)

subspecies ***cembrae*** (Cholodkovsky 1888)

=*sibiricus* (Cholodkovsky 1889b:388) (*Chermes*)

subspecies ***pinikoreanus***Zhang and Fang 1981:15 (subspecies of *Pineus cembrae* (Cholokovsky))

***cladogenous***Fang and Sun 1985:3 (*Pineus*)

***coloradensis*** (Gillette 1907:16) (*Chermes*)

***cortecicolus***Fang and Sun 1985:2 (*Pineus*)

***engelmannii***Annand 1928:36,130 (*Pineus*)

***floccus*** (Patch 1909:137) (*Chermes*)

***ghanii***Yaseen and Ghani 1971:191,199 (*Pineus*)

***harukawai***Inouye 1945:13,21,31,80 (*Pineus*)

***havrylenkoi***Blanchard 1944:52 (*Pineus*)

***hosoyai***Inouye 1945:22,32,81 (*Pineus*)

***konowashiyai***Inouye 1945:13,21,34,81 (*Pineus*)

***laevis*** (Maskell 1885:16) (variety of *Kermaphis pini* (Koch))

***matsumurai***Inouye 1945:13,22,37,82 (*Pineus*)

***orientalis*** (Dreyfus 1888:3,6) (*Chermes*)

***patchae***Börner 1926:239 (*Pineus*)

***pineoides*** (Cholodkovsky 1903:263) (variety of *Chermes pini* (Koch))

***pini*** (Goeze 1778:328) (*Chermes*)

=*coniferarum* (Cholodkovsky 1889a:222) (*Chermes*)

=*pini* (Koch 1857:322) (*Anisophleba*)

=*pinicola* (Cholodkovsky 1915:57) (variety of *Chermes pini* (Koch))

***piniyunnanensis***Zhang et al. 1992:361,394 (*Pineus*)

***sichunanus*** Zhang in Zhang et al. 1980:381 (*Pineus*)

***similis*** (Gillette 1907:15) (*Chermes*)

***simmondsi***Yaseen and Ghani 1971:191,195 (*Pineus*)

***strobi*** (Hartig 1839:643) (*Coccus*)

=*corticalis* (Kaltenbach 1843:197) (*Chermes*)

=*pinicorticis* (Fitch 1855:871) (*Coccus*)

=*strobi* (Baerensprung 1849:174) (*Coccus*)

=*strobi* (Ratzeburg 1844:203) (*Chermes*)

***sylvestris***Annand 1928:34,115 (*Pineus*)

***wallichianae***Yaseen and Ghani 1971:191,202 (*Pineus*)

### Index of genus-group and species-group names

***ADELGES*** Vallot 1836

*abieticolens* Thomas 1879 – synonym of Pineus (Pineodes) pinifoliae

***abietinus*** Underwood and Balch 1964 – Pineus (Pineus)

***abietis*** Linnaeus 1758 – Adelges (Sacchiphantes)

*abietislaricis* Eckstein 1890 – synonym of Adelges (Sacchiphantes) abietis

*abietispiceae* Stebbing 1903 – Adelges (Dreyfusia)

***aenigmaticus*** Annand 1928 – Adelges (Adelges)

*affinis* Börner 1908b – synonym of Adelges (Adelges) tardus

*alaeviridis* Solowiow 1924 – synonym of Adelges (Sacchiphantes) abietis

*ANISOPHLEBA* Koch 1857 – synonym of Adelges (Adelges)

***ANNANDINA*** Favret et al. 2015 – subgenus of *Adelges*

*APHANUS* Gistel 1837 – synonym of *Gisteliella*

***APHRASTASIA*** Börner 1909a – subgenus of *Adelges*

***armandicola*** Zhang et al. in Chen 1992 – Pineus (Pineus)

*armiger* Annand 1924a – synonym of Pineus (Pineodes) pinifoliae

*atratus* Buckton 1883 – synonym of Adelges (Adelges) laricis larici

***boerneri*** Annand 1928 – Pineus (Pineus)

*bouvieri* Cholodkovsky 1902 – synonym of Adelges (Dreyfusia) piceae piceae

***boycei*** Annand 1928 – Pineus (Pineus)

***canadensis*** Merker and Eichhorn 1956 – subspecies of Adelges (Dreyfusia) piceae

***cembrae*** Cholodkovsky 1888 – Pineus (Pineus)

*CHERMAPHIS* Maskell 1884 – synonym of Pineus (Pineus)

*CHERMES* Linnaeus 1758

***CHOLODKOVSKYA*** Börner 1909b – subgenus of *Adelges*

***cladogenous*** Fang and Sun 1985 – Pineus (Pineus)

*CNAPHALODES* Amyot and Audinet Serville 1843 – synonym of Pineus (Pineus)

*coccineus* Ratzeburg 1843 – synonym of Adelges (Adelges) laricis laricis

***coloradensis*** Gillette 1907 – Pineus (Pineus)

*coniferarum* Cholodkovsky 1889a – synonym of Pineus (Pineus) pini

*consolidatus* Patch 1909 – synonym of Adelges (Adelges) laricis laricis

***cooleyi*** Gillette 1907 – Adelges (Gilletteella)

***cortecicolus*** Fang and Sun 1985 – Pineus (Pineus)

*corticalis* Kaltenbach 1843 – synonym of Pineus (Pineus) strobi

***coweni*** Gillette 1907 – Adelges (Gilletteella)

*cummingae* Steffan 1968 – Adelges (Gilletteella)

***diversis*** Annand 1928 – Adelges (Sacchiphantes)

***DREYFUSIA*** Börner 1908a – subgenus of *Adelges*

*ELATIPTUS* Amyot 1847 – synonym of Adelges (Sacchiphantes)

***engelmannii*** Annand 1928 – Pineus (Pineus)

*EOPINEUS* Steffan 1968 – synonym of Pineus (Pineus)

***floccus*** Patch 1909 – Pineus (Pineus)

*funitectus* Dreyfus 1888 – Adelges (Dreyfusia)

*gallarumabietis* De Geer 1773 – synonym of Adelges (Sacchiphantes) abietis

***geniculatus*** Ratzeburg 1844 – Adelges (Adelges)

***ghanii*** Yaseen and Ghani 1971 – Pineus (Pineus)

*GILLETTEA* Börner 1909d – synonym of Adelges (Gilletteella)

***GILLETTEELLA*** Börner 1930 – subgenus of *Adelges*

*GISTELIELLA* Strand 1928

***glandulae*** Zhang in Zhang et al. 1980 – Adelges (Gilletteella)

*hamadryas* Koch 1857 – synonym of Adelges (Adelges) laricis laricis

***harukawai*** Inouye 1945 – Pineus (Pineus)

***havrylenkoi*** Blanchard 1944 – Pineus (Pineus)

*himalayensis* Stebbing 1910 – synonym of Adelges (Dreyfusia) abietispiceae

***hosoyai*** Inouye 1945 – Pineus (Pineus)

***isedakii*** Eichhorn in Eichhorn and Carter 1978 – Adelges (Adelges)

***ishiharai*** Inouye 1936 – subspecies of Adelges (Aphrastasia) pectinatae

***japonicus*** Monzen 1929 – Adelges (Adelges)

***joshii*** Schneider-Orelli and Schneider 1959 – Adelges (Dreyfusia)

***karafutonis*** Kono and Inouye 1938 – Adelges (Sacchiphantes)

***karamatsu*** Inouye 1945 – Adelges (Adelges)

*KERMAPHIS* Maskell 1885 – synonym of Pineus (Pineus)

***kitamiensis*** Inouye 1963 – Adelges (Sacchiphantes)

***knucheli*** Schneider-Orelli and Schneider 1954 – Adelges (Dreyfusia)

***konowashiyai*** Inouye 1945 – Pineus (Pineus)

***laevis*** Maskell 1885 – Pineus (Pineus)

*lapidaria* Fabricius 1803 – *Gisteliella*

***lapponicus*** Cholodkovsky 1889b – Adelges (Adelges)

*LARICETHUS* Amyot 1847 – synonym of Adelges (Adelges)

*lariceti* Altum 1889 – synonym of Adelges (Adelges) laricis laricis

***lariciatus*** Patch 1909 – Adelges (Adelges)

*laricicola* Shinji 1930 – synonym of Adelges (Cholodkovskya) viridanus

*laricifoliae* Fitch 1858 – synonym of Adelges (Sacchiphantes) abietis

*laricis* Bouché 1834 – synonym of Adelges (Adelges) laricis laricis

*laricis* Hartig 1839 – synonym of Adelges (Adelges) laricis laricis

***laricis*** Vallot 1836 – Adelges (Adelges)

***matsumurai*** Inouye 1945 – Pineus (Pineus)

***merkeri*** Eichhorn 1957 – Adelges (Dreyfusia)

*montanus* Gillette 1907 – synonym of Pineus (Pineodes) pinifoliae

***nebrodensis*** Binazzi and Covassi 1991 – Adelges (Dreyfusia)

*niger* Solowiow 1924 – synonym of Adelges (Adelges) tardus

***nordmannianae*** Eckstein 1890 – Adelges (Dreyfusia)

*nuesslini* Börner 1908c – synonym of Adelges (Dreyfusia) nordmannianae

*obtectus* Ratzeburg 1844 – synonym of Adelges (Adelges) laricis laricis

*occidentalis* Cholodkovsky 1910 – synonym of Adelges (Sacchiphantes) viridis

***occidentalis*** Foottit and Mackauer 1983 – subspecies of Adelges (Dreyfusia) piceae

***oregonensis*** Annand 1928 – Adelges (Cholodkovskya)

***orientalis*** Dreyfus 1888 – Pineus (Pineus)

***patchae*** Börner 1926 – Pineus (Pineus)

***pectinatae*** Cholodkovsky 1888 – Adelges (Aphrastasia)

*PHLOEOPHTHIRIDIUM* van der Hoeven 1849 – synonym of Adelges (Sacchiphantes)

***piceae*** Ratzeburg 1944 – Adelges (Dreyfusia)

***pindrowi*** Yaseen and Ghani 1971 – Adelges (Dreyfusia)

***PINEODES*** Börner 1926 – subgenus of *Pineus*

***pineoides*** Cholodkovsky 1903 – Pineus (Pineus)

***PINEUS*** Shimer 1869

***pini*** Goeze 1778 – Pineus (Pineus)

*pini* Koch 1857 – synonym of Pineus (Pineus) pini

*pinicola* Cholodkovsky 1915 – synonym of Pineus (Pineus) pini

*pinicorticis* Fitch 1855 – synonym of Pineus (Pineus) strobi

***pinifoliae*** Fitch 1858 – Pineus (Pineodes)

***pinikoreanus*** Zhang and Fang 1981 – subspecies of Pineus (Pineus) cembrae

***piniyunnanensis*** Zhang et al. 1992 – Pineus (Pineus)

*PITYOPSYLLA* Amyot 1847 – synonym of Pineus (Pineus)

***potaninilaricis*** Zhang in Zhang et al. 1980 – subspecies of Adelges (Adelges) laricis

*praecox* Cholodkovsky 1898 – synonym of Adelges (Adelges) lapponicus

***prelli*** Grosmann 1935 – Adelges (Dreyfusia)

***roseigallis*** Li and Tsai 1973 – Adelges (Sacchiphantes)

***SACCHIPHANTES*** Curtis 1844 – subgenus of *Adelges*

***schneideri*** Börner 1931 – Adelges (Dreyfusia)

***segregis*** Steffan 1961 – Adelges (Sacchiphantes)

*sibiricus* Cholodkovsky 1889b – synonym of Pineus (Pineus) cembrae cembrae

***sichunanus*** Zhang in Zhang et al. 1980 – Pineus (Pineus)

***similis*** Gillette 1907 – Pineus (Pineus)

***simmondsi*** Yaseen and Ghani 1971 – Pineus (Pineus)

*strobi* Baerensprung 1849 – synonym of Pineus (Pineus) strobi

***strobi*** Hartig 1839 – Pineus (Pineus)

*strobi* Ratzeburg 1844 – synonym of Pineus (Pineus) strobi

*strobilobius* Kaltenbach 1843 – synonym of Adelges (Adelges) laricis laricis

***sylvestris*** Annand 1928 – Pineus (Pineus)

***tardoides*** Cholodkovsky 1911 – Adelges (Adelges)

***tardus*** Dreyfus 1888 – Adelges (Adelges)

***todomatsui*** Inouye 1945 – Adelges (Dreyfusia)

***torii*** Eichhorn in Eichhorn and Carter 1978 – Adelges (Sacchiphantes)

***tsugae*** Annand 1924b – Adelges (Annandina)

***viridanus*** Cholodkovsky 1896 – Adelges (Cholodkovskya)

***viridis*** Ratzeburg 1843 – Adelges (Sacchiphantes)

***viridulus*** Cholodkovsky 1911 – Adelges (Cholodkovskya)

***wallichianae*** Yaseen and Ghani 1971 – Pineus (Pineus)

## References

[B1] Altum B (1889) Waldbeschädigungen durch Thiere und Gegenmittel. Julius Springer, Berlin, 285 pp. doi: 10.1007/978-3-642-50828-8

[B2] Amyot CJB (1847) Entomologie française. Rhynchotes (Suite) (1). Annales de la Société Entomologique de France. Deuxième Série 5: 453–506.

[B3] Amyot CJB Audinet Serville JG (1843) Histoire Naturelle des Insectes. Hémiptères. Librairie encyclopédique de Roret, Paris, 675 pp.

[B4] Annand PN (1924a) A new *Chermes* from pine (Hemiptera Aphidae). Canadian Entomologist 56(1): 5–6. doi: 10.4039/Ent565-1

[B5] Annand PN (1924b) A new species of *Adelges* (Hemiptera, Phylloxeridae). Pan-Pacific Entomologist 1(2): 79–82.

[B6] Annand PN (1928) A contribution toward a monograph of the Adelginae (Phylloxeridae) of North America. Stanford University Publication, University Series, Biological Sciences 6(1): 1–146.

[B7] Ashmead WH (1897) Description of some new genera in the family Cynipidae. Psyche 8(253): 67–69. doi: 10.1155/1897/60546

[B8] Baerensprung FV (1849) Beobachtungen über einige einheimische Arten aus der Familie der Coccinen. Zeitung für Zoologie, Zootomie und Palaeozoologie 1(22): 173–176.

[B9] Battisti A Binazzi A Gourov A Khomentovsky P Roques A (1997) A survey of conifer aphids from Siberia and Kamchatka (Homoptera Aphidoidea). Redia 80: 131–145.

[B10] Binazzi A (1984) Chiave per le specie afidiche più note delle conifere in Europa. Redia 67(appendix): 547–571.

[B11] Binazzi A Covassi M (1991) Contributi alla conoscenze degli afidi delle conifere XII. Il gen. *Dreyfusia* Boerner in Italia con la descrizione di una specia nuova (Homoptera Adelgidae). Redia 74(1): 233–299.

[B12] Blanchard EE (1944) Descripciones y anotaciones de afidoideos argentinos. Acta Zoologica Lilloana 2: 15–62.

[B13] Börner C (1908a) [1907] Systematik und Biologie der Chermiden. Zoologischer Anzeiger 32(14): 413–428.

[B14] Börner C (1908b) Eine monographische Studie über die Chermiden. Arbeiten aus der Kaiserlichen Biologischen Anstalt für Land- und Forstwirtschaft 6(2): 81–320.

[B15] Börner C (1908c) Über Chermesiden IV. *Dreyfusia piceae* und *nüsslini* nov. spec. Zoologischer Anzeiger 33(22/23): 737–750.

[B16] Börner C (1909a) *Aphrastasia pectinatae* (Chol.) CB. Self-published, Sankt Julian bei Metz, 1 pp.

[B17] Börner C (1909b) *Cholodkovskya viridana* (Chol.) CB. Self-published, Sankt Julian bei Metz, 2 pp.

[B18] Börner C (1909c) Untersuchungen über die Chermiden. Mitteilungen aus der Kaiserlichen Biologischen Anstalt für Land- und Forstwirtschaft 8: 50–60.

[B19] Börner C (1909d) Über Chermesiden VI. *Cholodkovskya*, *Aphrastasia* und *Gillettea*. Zoologischer Anzeiger 34(16/17): 498–511.

[B20] Börner C (1926) Zuchtung der Homopteren. In: Abderhalden E (Ed.) Handbuch der biologischen Arbeitsmethoden. 9 part 1(2) Urban & Schwarzenberg, Berlin & Wien, 215–270.

[B21] Börner C (1930) Beiträge zu einem neuen System der Blattläuse. Archiv für klassifikatorische und phylogenetische Entomologie 1(2): 115–180.

[B22] Börner C (1931) Aphidoidea, Blattläuse. Sorauer Handbuch der Pflanzenkrankheiten [offprint]. [See Nieto Nafría et al. 2007: 54]

[B23] Börner C (1952) Europae centralis Aphides. Die Blattläuse Mitteleuropas. Namen, Synonyme, Wirtspflanzen, Generationszyklen. Mitteilungen der Thüringischen Botanischen Gesellschaft, Supplement 3: 1–259.

[B24] Bouché PF (1834) Naturgeschichte der Insekten, besonders in Hinsicht ihrer ersten Zustände als Larven und Puppen. Nicolaischen Buchhandlung, Berlin, 216 pp.

[B25] Buckton GB (1883) Monograph of British Aphides. Volume 4 Ray Society, London, 228 pp.

[B26] Carter CI (1971) Conifer woolly aphids (Adelgidae) in Britain. Forestry Commission Bulletin 42: 1–51.

[B27] China WE (Ed.) (1963) Opinion 686. Amyot, Méthode mononymique: placed on the official index of rejected and invalid works in zoological nomenclature. Bulletin of Zoological Nomenclature 20(6): 423.

[B28] Cholodkovsky NA (1888) Über einige *Chermes*-Arten. Zoologischer Anzeiger 11(270): 45–48.

[B29] Cholodkovsky NA (1889a) Weiteres zur Kenntnis der *Chermes*-Arten. Zoologischer Anzeiger 12(305): 218–223.

[B30] Cholodkovsky NA (1889b) Neue Mittheilungen zur Lebensgeschichte der Gattung *Chermes* L. Zoologischer Anzeiger 12(312): 387–391.

[B31] Cholodkovsky NA (1896) Zur Biologie der Lärchen-*Chermes*-Arten. Zoologischer Anzeiger 19(494): 37–40.

[B32] Cholodkovsky NA (1898) Beiträge zu einer Monographie der Coniferen-Läuse. Trudy Vsesojuznogo Entomologiceskogo Obscestva 31: 1–61.

[B33] Cholodkovsky NA (1902) Second annotated catalogue of aphid (Aphidae) collections in Zoological Cabinet of St. Petersburg Institute of Forestry. Izvestiya S-Peterburgskogo Lesnogo Instituta 8(2): 1–11.

[B34] Cholodkovsky NA (1903) Aphidologische Mittheilungen. Zoologischer Anzeiger. 26(693): 258–263.

[B35] Cholodkovsky NA (1906) *Chermes* injurious to coniferous trees. Department of Agriculture, St. Petersburg, 60 pp.

[B36] Cholodkovsky NA (1910) Aphidologische Mitteilungen. Zoologischer Anzeiger 35(9/10): 279–284.

[B37] Cholodkovsky NA (1911) Aphidologische Mitteilungen. Zoologischer Anzeiger 37(8/9): 172–178.

[B38] Cholodkovsky NA (1915) *Chermes* injurious to coniferous trees (2). Department of Agriculture, Petrograd, 89 pp.

[B39] Cortés Gabaudan F Nieto Nafría JM Favret C Barbagallo S Sano M Stekolshchikov AV (2011) Etymology and gender of genus-group names. In: Nieto Nafría JM Favret C (Eds) Registers of Family-Group and Genus-Group Taxa of Aphidoidea (HemipteraSternorrhyncha). Universidad de León, León, 405–463.

[B40] Curtis J (1844) The spruce-cone Gall *Aphis* – (*Sacchiphantes Abietis*). The Gardeners’ Chronicle and Agricultural Gazette 50: 831.

[B41] De Geer C (1773) Mémoires pour Servir à l’Histoire des Insectes. Volume 3. Pierre Hesselberg, Stockholm, 696 pp.

[B42] Dreyfus LT (1888) Über neue Beobachtungen bei den Gattungen *Chermes* und *Phylloxera*. Versammlung deutscher Naturforscher und Ärzte zu Köln 61: 1–10.

[B43] Dreyfus LT (1889) Ueber Phylloxerinen. J.F. Bergman, Wiesbaden, 88 pp. doi: 10.5962/bhl.title.8560

[B44] Eastop VF Hille Ris Lambers D (1976) Survey of the World’s Aphids. Dr. W. Junk, The Hague, 573 pp.

[B45] Eckstein K (1890) Zur Biologie der Gattung *Chermes* L. Zoologischer Anzeiger 13(328): 86–90.

[B46] Eichhorn O Carter CI (1978) Investigation into conifer woolly aphids (Hemiptera: Adelgidae) in Japan, with the description of two new species. Zeitschrift für angewandte Entomologie 86: 273–289. doi: 10.1111/j.1439-0418.1978.tb01934.x

[B47] Eichhorn O (1957) Eine neue Tannenlaus der Gattung *Dreyfusia* (*Dreyfusia merkeri* nov. spec.). Zeitschrift für Angewandte Zoologie 44(3): 303–348.

[B48] Eichhorn O (1994) Numerical research upon the types and rates of offspring produced by the parthenogenetic generations of *Adelges laricis* Vall. (Hom., Adelgidae). Journal of Applied Entomology 117(1–5): 344–352. doi: 10.1111/j.1439-0418.1994.tb00745.x

[B49] Evans GO China WE (Eds) (1965) Opinion 731. *Psylla* Geoffroy, 1762 (Insecta, Hemiptera): validated under the plenary powers with suppression of *Chermes* Linnaeus, 1758. Bulletin of Zoological Nomenclature 22(2): 86–87.

[B50] Fabricius JC (1803) Systema Rhyngotorum. Secundum Ordines, Genera, Species. Adiectis Synonymis, Locis, Observationibus, Descriptionibus. Carolum Reichard, Brunsvigae (Brunswick), 314 pp. doi: 10.5962/bhl.title.11644

[B51] Fang SY (1982) Biological statistical information on wing vein patterns of six aphids in the Xiaoxinanlin area. Journal of North-East Forestry University 6: 24–26.

[B52] Fang SY Yan SC (1997) The conifer woolly aphids (Adelgidae) in China. Journal of Forestry Research 8(1): 30–32. doi: 10.1007/BF02864936

[B53] Fang SY Sun JH (1985) Study of Korean pine adelgids in Northeast China, with descriptions of two new species. Journal of North-East Forestry University 13(3): 1–7.

[B54] Favret C Miller GL Nieto Nafría JM Cortés Gabaudan F (2008) [2007] Catalog of the aphid genera described from the New World. Transactions of the American Entomological Society 133(3–4): 363–412.

[B55] Favret C Miller GL Nieto Nafría JM Cortés Gabaudan F (2009) [2008]. Corrections and additions to the catalog of the aphid genera described from the New World. Transactions of the American Entomological Society 134(3–4): 275–282.

[B56] Favret C Ouvrard D Williams DJ (2014) Annotated list of the species-group taxa described in combination with *Chermes* Linnaeus 1758 (Hemiptera: Sternorrhyncha). Transactions of the American Entomological Society 140: 67–81. doi: 10.3157/061.140.0104

[B57] Favret C Blackman RL Stekolshchikov AV (2015) *Chermes funitectus* Dreyfus and the hemlock woolly adelgid (Hemiptera: Adelgidae). Entomological News 125(3): 174–178. doi: 10.3157/021.125.0304

[B58] Fitch A (1855) [1854]. Report on the noxious, beneficial, and other insects of the state of New York. Transactions of the New York State Agricultural Society 14: 691–880.

[B59] Fitch A (1858) [1857]. Fourth report on the noxious and other insects of the State of New York. Transactions of the New York State Agricultural Society 17: 687–814.

[B60] Foottit R Mackauer M (1983) Subspecies of the balsam woolly aphid, *Adelges piceae* (Homoptera: Adelgidae), in North America. Annals of the Entomological Society of America 76(2): 299–304. doi: 10.1093/aesa/76.2.299

[B61] Gavrilov-Zimin IA Stekolshchikov AV Gautam DC (2015) General trends of chromosomal evolution in Aphidococca (Insecta, Homoptera, Aphidinea + Coccinea). Comparative Cytogenetics 9(3): 335–422. doi: 10.3897/CompCytogen.v9i3.4930 26312130 10.3897/CompCytogen.v9i3.4930 PMC4547034

[B62] Ghosh AK (1983) A review of the family Adelgidae from the Indian subregion (Homoptera: Aphidoidea). Oriental Insects 17(1): 1–34. doi: 10.1080/00305316.1983.10433690

[B63] Gillette CP (1907) *Chermes* of Colorado conifers. Proceedings of the Academy of Natural Sciences of Philadelphia, 54(1): 3–22.

[B64] Gistel J (1837) Systematische Uebersicht der Wanzen und Cicaden der Umgebung von München. Faunus. Zeitschrift für Zoologie und vergleichende Anatomie 21: 98–111.

[B65] Goeze JAE (1778) Entomologische Beyträge zu des Ritter Linné Zwölften Ausgabe des Natursystems. Volume 2. Weidmann Erben und Reich, Leipzig, 352 pp.

[B66] Grosmann H (1935) Über eine neue Tannenlaus (*Dreyfusia Prelli* nov. spec.). Tharandter Forstliches Jahrbuch 86: 816–826.

[B67] Hartig T (1839) Jahresberichte Über die Fortschritte der Firstwissenschaft und forstlichen Naturkunde im Jahre 1836 und 1837 nebst Original-Abhandlungen aus dem Gebiete dieser Wissenschaften 1(4): 489–646.

[B68] Havill NP Foottit RG (2007) Biology and evolution of Adelgidae. Annual Review of Entomology 52: 325–349. doi: 10.1146/annurev.ento.52.110405.091303 10.1146/annurev.ento.52.110405.091303 17163799

[B69] Havill NP Foottit RG von Dohlen CD (2007) Evolution of host specialization in the Adelgidae (Insecta: Hemiptera) inferred from molecular phylogenetics. Molecular Phylogenetics and Evolution 44: 357–370. doi: 10.1016/j.ympev.2006.11.008 17196838 10.1016/j.ympev.2006.11.008

[B70] Heie OE Wegierek P (2009) A classification of the Aphidomorpha (Hemiptera Sternorrhyncha) under consideration of the fossil taxa. Redia 92: 69–77.

[B71] Heie OE Wegierek P (2011) A list of fossil aphids (Hemiptera, Sternorrhyncha, Aphidomorpha). Monographs of the Upper Silesian Museum 6, 82 pp.

[B72] Herrich-Schaeffer D (1854) Vorwort des Herausgebers. In: Koch CL, Die Pflanzenläuse Aphiden getreu nach dem Leben abgebildet und beschrieben. J.L. Lotzbeck, Nürnberg Volume 1: III-VIII.

[B73] Hunter WD (1901) The Aphididae of North America. Bulletin of the Iowa Agricultural Experiment Station 60: 63–138.

[B74] Inouye M (1936) Eine neue *Chermes*- Art (Adelgiden) auf Hokkaido. Insecta Matsumurana 11(1–2): 75–80.

[B75] Inouye M (1945) Monographische Studie über die Japanischen Koniferen-Gallenläuse (Adelgidae). Bulletin of the Hokkaido Forestry Experiment Station 15, 105 pp.

[B76] Inouye M (1963) Eine neue *Sacchiphantes*-Art von Adeligen, Schädlinge an Japanlärchen in Japan. Journal of the Japanese Forestry Society 45(10): 340–344.

[B77] International Commission on Zoological Nomenclature (ICZN) (1999) International Code of Zoological Nomenclature, Fourth Edition. International Trust for Zoological Nomenclature, London, 306 pp.

[B78] Kaltenbach JH (1843) Monographie der Familien der Pflanzenläuse (Phytophthires). P. Fagot, Aachen, 222 pp. doi: 10.5962/bhl.title.51483

[B79] Koch CL (1857) Die Pflanzenläuse Aphiden getreu nach dem Leben abgebildet und beschrieben. J.L. Lotzbeck, Nürnberg Volume 9: 275–335.

[B80] Kono H Inouye M (1938) Die Adelgiden, schädlich an Ezofichten und Akaezofichten in Japan. Insecta Matsumurana 12(4): 169–173.

[B81] Laporte FL (1833) [1832] Essai d’une classification systématique de l’ordre des Hémiptères (Hémiptères Héteroptères, Latr.). Magasin de Zoologie 2: 1–88.

[B82] Li CL Tsai PH (1973) Studies on the larch adelgids in northeast China with description of a new species. Acta Entomologica Sinica 16(2): 133–153.

[B83] Linnaeus C (1758) Systema naturae per regna tri naturae, secundum classes, ordines, genera, species, cum characteribus, differentiis, synonymis, locis. Editio Decima, Reformata. Volume 1 Laurentii Salvii, Holmiae (Stockholm), 824 pp.

[B84] Luo Y (1988) Life cycle and control of *Adelges laricis* Vall in the forestry areas of Liupan mountains. Ningxia Journal of Agro-forestry Science and Technology (China) 4: 23–24.

[B85] Maskell WM (1884) On an aphidian insect infesting pine trees. New Zealand Journal of Science 2(6): 291–292.

[B86] Maskell WM (1885) Note on an aphidian insect infesting pine trees, with observations on the name “*Chermes*” or “*Kermes*”. Transactions of the New Zealand Institute 17: 13–19.

[B87] Merker E Eichhorn O (1956) Die Formenkreise der gefährlichen Tannenläuse Gattung Dreyfusia (Adelges). Naturwissenschaften 19(43): 453–454. doi: 10.1007/BF00629526

[B88] Michalik A Szklarzewicz T Wegierek P Wieczorek K (2013) The ovaries of aphids (Hemiptera, Sternorrhyncha, Aphidoidea): morphology and phylogenetic implications. Invertebrate Biology 132(3): 226–240. doi: /10.1111/ivb.12026

[B89] Monzen K (1929) Studies on some gall producing aphids and their galls. Saito Ho-on Kai (The Saito Gratitude Foundation) Monographs 1. The General Department of Scientific Research, Saito Ho-on Kai, Sendai, Japan, 80 pp.

[B90] Nieto Nafría JM Favret C Akimoto S Barbagallo S Chakrabarti S Mier Durante MP Miller GL Qiao GX Sano M Pérez Hidalgo N Stekolshchikov AV Wegierek P (2011) Register of genus-group taxa of Aphidoidea. In: Nieto Nafría JM Favret C (Eds) Registers of Family-Group and Genus-Group Taxa of Aphidoidea (HemipteraSternorrhyncha). Universidad de León, León, 81–404.

[B91] Nieto Nafría JM Pérez Hidalgo N Mier Durante MP (2007) New synonyms and several nomenclatural clarifications on family-group names in the Aphididae (Hemiptera Sternorrhyncha). Zootaxa 1629: 51–55.

[B92] Parry WH (1973) Observations on the flight periods of aphids in a Sitka spruce plantation in north-eastern Scotland. Bulletin of Entomological Research 62(3): 391–399. doi: 10.1017/S0007485300003904

[B93] Patch EM (1909) *Chermes* of Maine spruces. Psyche 16: 136–137. doi: 10.1155/1909/942383

[B94] Patch EM (1938) Food-plant catalogue of the aphids of the world including the Phylloxeridae. Maine Agricultural Experiment Station Bulletin 393: 36–431.

[B95] Podeur G (1971) Contributions to the study of the diapause of certain Adelgidae (Homoptera: Aphidoidea) in the Lorraine region. Bulletin de l’École Nationale Superieure Agronomique de Nancy 13: 50–54.

[B96] Ratzeburg JTC (1843) Bericht über einige neue, den Waldbäumen schädliche Rhynchoten. Entomologische Zeitung 4(7): 201–204.

[B97] Ratzeburg JTC (1844) Die Forst-Insecten oder Abbildung und Beschreibung der in den wäldern Preussens und der Nachbarstaaten als schädlich oder nützlich bekannt gewordenen Insecten: Im systematischer Folge und mit besonderer Rücksicht auf die Vertilgung der Schädlichen. Volume 3. Nicolaische buchhandlung, Berlin, 314 pp.

[B98] Remaudière G Remaudière M (1997) Catalogue of the World’s Aphididae. INRA, Paris, 473 pp.

[B99] Rohfritsch O (1988) A resistance response of *Picea excelsa* to the aphid, (Homoptera: Aphidoidea) *Adelges abietis*. In: Mattson WJ Levieux J Bernard-Daga C (Eds) Mechanisms of Woody Plant Defenses Against Insects. Springer, New York, 253–266. doi: 10.1007/978-1-4612-3828-7_16

[B100] Rożkowski R (2004) Breeding value of Norway sprunce (*Picea abies* [L.] Karst.) from the Kłodzko Forest District (SW Poland). Journal of Forest Science 50(1): 17–23.

[B101] Sano M Ozaki K (2012) Variation and evolution of the complex life cycle in Adelgidae (Hemiptera). Entomological Science 15: 13–22. doi: 10.1111/j.1479-8298.2011.00483.x

[B102] Schneider-Orelli O Schneider F (1954) *Dreyfusia*-Befall an *Abies pindrow* im Nordwesthimalaya. Mitteilungen der Schweizerischen Entomologischen Gesellschaft 27(4): 413–422.

[B103] Schneider-Orelli O Schneider F (1959) Weitere Untersuchungen über *Dreyfusia*-Befall im Nordwesthimalaya (Homopt. Adelgidae). Mitteilungen der Schweizerischen Entomologischen Gesellschaft 32(2–3): 258–270.

[B104] Shimer H (1869) [1868] Notes on *Chermes pinicorticis*. Transactions of the American Entomological Society 2(1): 383–385.

[B105] Shinji GO (1930) Some new Aphidids from North Eastern Japan. Lansania 2(20): 151–160.

[B106] Skrzypczyńska M (2004) Relationships between the number of dwarf shoots of *Larix decidua* Mill. damaged by different insect species in the Ojców National Park in southern Poland. Journal of Pest Science 77(3): 173–177. doi: 10.1007/s10340-004-0051-8

[B107] Solowiow P (1924) Beobachtungen über neue Arten der Gattung Chermes: *Chermes alaeviridis* n. sp. und *Chermes niger* n. sp. (Rhynchota, Phytophthires, Aphidae, Phylloxeridae). Zoologischer Anzeiger 60: 38–49.

[B108] Stebbing EP (1903) [1904] On the acquisition of alar appendages by the spruce form of *Chermes abeitis-piceae* MS. in the N.-W. Himalayas. Journal of the Asiatic Society of Bengal 72(2): 57–60.

[B109] Stebbing EP (1910) On the life-history of *Chermes himalayensis*, Steb., on the spruce (*Picea morinda*) and silver fir (*Abies webbiana*). Transactions of the Linnean Society of London. Second series 11(Part 6): 99–124.

[B110] Steffan AW (1961) Die Stammes- und Siedlungsgeschichte des Artenkreises *Sacchiphantes viridis* (Ratzeburg 1843) (Adelgidae, Aphidoidea). Zoologica: Original Abhandlungen aus dem Gesamtgebiete der Zoologie 109, 113 pp.

[B111] Steffan AW (1968) Evolution und Systematik der Adelgidae (Homoptera: Aphidina). Eine Verwandtschaftsanalyse auf vorwiegend ethologischer, zytologischer und kardiologischer Grundlage. Zoologica, Original-Abhandlungen aus dem Gesamtgebiete der Zoologie. 40(lieferung 5 heft 115), 139 pp.

[B112] Steffan AW (1972) Unterordnung Aphidina, Blattläuse. In: Schwenke W (Ed.) Die Forstschädlinge Europas. Volume 1 Paul Parey, Hamburg, 162–386.

[B113] Strand E (1928) [1926] Miscellanea nomenclatorica zoologica et palaeontologica. I-II. Archiv für Naturgeschichte 92(A-8): 30–75.

[B114] Szklarzewicz T Wnęk A Biliński SM (2000) Structure of ovarioles in *Adelges laricis*, a representative of the primitive aphid family Adelgidae. Acta Zoologica 81(4): 307–313. doi: 10.1046/j.1463-6395.2000.00061.x

[B115] Takahashi R (1937) Phylloxeridae of Formosa (Hemiptera). Transactions of the Natural History Society of Formosa 27(160): 11–14.

[B116] Thomas C (1879) Third annual report by Cyrus Thomas. Report of the State Entomologist on the Noxious and Beneficial Insects of the State of Illinois, 8th report, 212 pp.

[B117] Toenshoff ER Daniela G Horn M (2012) Co-evolution and symbiont replacement shaped the symbiosis between adelgids (Hemiptera: Adelgidae) and their bacterial symbionts. Environmental Microbiology 14(5): 1284–1295. doi: 10.1111/j.1462-2920.2012.02712.x 22364314 10.1111/j.1462-2920.2012.02712.x

[B118] Underwood GR Balch RE (1964) A new species of *Pineus* (Homoptera: Adelgidae) on *Abies*. Canadian Entomologist 96(3): 522–528. doi: 10.4039/Ent96522-3

[B119] Vallot JN (1836) Observations sur deux insectes hémiptères qui vivent, l’un sur le mélèze et l’autre sur le cafier. Comptes rendus hebdomadaires des Séances de l’Académie des Sciences 3: 72–73.

[B120] van der Hoeven J (1849) Handboek der Dierkunde. Volume 1 C.G. Sulpke, Amsterdam, 791 pp.

[B121] Wegierek P (2002) Relationships within Aphidomorpha on the basis of thorax morphology. University of Silesia, Katowice, Poland, 107 pp.

[B122] Wegierek P (2003) Apterous Phylloxeroidea (Hemiptera, Sternorrhyncha) from Baltic amber. Acta Zoologica Cracoviensia 46(fossil insects): 277–283.

[B123] Wilson HF Vickery RA (1918) A species list of the Aphididae of the world and their recorded food plants. Transactions of the Wisconsin Academy of Sciences, Arts, and Letters 19: 22–352.

[B124] Yaseen M Ghani MA (1971) Descriptions, and notes on the biology, of four new species of Adelgidae from West Pakistan. Bulletin of Entomological Research 61: 191–205. doi: 10.1017/S0007485300057722

[B125] Zhang GX Fang SY (1981) A description of *Pineus cembrae pinikoreanus* Zhang et Fang, subsp. nov. (Homoptera: Adelgidae). Journal of North-East Forestry University 12(4): 15–18.

[B126] Zhang GX Zhong TS Tian ZJ (1980) Two new species and a new subspecies of Adelgidae from Sichuan China (Homoptera, Adelgidae). Zoological Research 1(3): 381–388.

[B127] Zhang GX Zhong TS Zhang WY (1992) Homoptera: Aphidoidea. In: Chen SX (Ed.) Insects of the Hengduan Mountains Region. Volume 1 Science Press, Beijing, 360–403.

[B128] Zurovcová M Havelka J Starý P Věchtová P Chundelová D Jarošová A Kučerová L (2010) “DNA barcoding” is of limited value for identifying adelgids (Hemiptera:Adelgidae) but supports traditional morphological taxonomy. European Journal of Entomology 107: 147–156. doi: 10.14411/eje.2010.020

